# Case conference primary-secondary care planning at end of life can reduce the cost of hospitalisations

**DOI:** 10.1186/s12904-016-0157-9

**Published:** 2016-09-23

**Authors:** Samantha Hollingworth, Jianzhen Zhang, Bharat Phani Vaikuntam, Claire Jackson, Geoffrey Mitchell

**Affiliations:** 1 School of Pharmacy, The University of Queensland, Woolloongabba, QLD 4102 Australia; 2 School of Medicine, The University of Queensland, Herston, QLD 4006 Australia

**Keywords:** Palliative care, Hospitalisation, Primary health care, Cost savings, Delivery of health care, Integrated

## Abstract

**Background:**

To plan integrated care at end of life for people with either heart failure or lung disease, we used a case conference between the patient’s general practitioner (GP), specialist services and a palliative care consultant physician. This intervention significantly reduced hospitalisations and emergency department visits. This paper reports estimates of potential savings of reduced hospitalisation through end of life case conferences in a pilot study.

**Methods:**

We used Australian Refined Diagnosis Related Group codes to obtain data on hospitalisations and costs. The Australian health system is a federation: the national government is responsible for funding community based care, while state and territory governments fund public hospitals. There were 35 case conferences for patients with end stage heart failure or lung disease, who were patients of the public hospital system, involving 30 GPs in a regional health district.

**Results:**

The annualised total cost per patient was AUD$90,060 before CC and AUD$11,841 after CC. The mean per person cost saving was AUD$41,023 ($25,274 excluding one service utilisation outlier). For every 100 patients with end of life heart failure and lung disease each year, the case conferencing intervention would save AUD$4.1 million (AUD$2.5 million excluding one service utilisation outlier).

**Conclusions:**

Multidisciplinary case conferences that promote integrated care among specialists and GPs resulted in substantial cost savings while providing care. Cost shifting between national and state or territory governments may impede implementation of this successful health service intervention. An integrated model such as ours is very relevant to initiatives to reform national health care.

**Trial registration:**

Australian and New Zealand Controlled Trials Register ACTRN12613001377729: Registered 16/12/2013.

## Background

Many palliative care services are offered to cancer patients [1] but most people die from non-malignant disease [2]. We advocate for an integrated model of care where primary care professionals including general practitioners (GPs) work with medical specialists to manage chronic diseases including palliative care [3] and diabetes [4, 5]. We conducted a pilot study of an integrated primary-secondary model of care consisting of a single case conference between GPs, disease-based specialist teams, and a palliative care consultant physician to plan the end of life care of people with end stage heart failure and lung disease [6]. We published results for the first 23 case conferences: annual rates of admissions to the emergency department (ED) decreased by 85 % to 2.1 per annum (pa; difference 11.8, 95 % CI 2.2; 21.3, *p* = 0.001) [6]. ED admissions leading to discharge home decreased from 3.9 to 0.4 pa (difference 3.5, 95 % CI −0.4; 7.5, *p* = 0.05); hospital admissions decreased 69 % from 11.4 to 3.5 pa (difference 7.9, 95 % CI 2.2; 13.7, *p* = 0.002); and length of stay decreased from 7.0 to 3.7 days (difference 3.4, 95 % CI 0.9; 5.8, *p* = 0.007). Furthermore, the participating health professionals were enthusiastic about the process [6]. Patients and their carers readily agreed to have their cases discussed in this way and appreciated the improvements in care they noticed (unpublished data). This paper reports the estimated cost savings associated with these reduced hospitalisations from case conferences in this integrated model of care.

Australia is a federation of states with overlapping, but separate responsibilities. It has a federally administered universal health insurance scheme (Medicare) which covers much of the cost of services by medical practitioners [7]. Medicare meets most of the costs for all out-of-hospital medical services, such as GP visits and specialist consultations.

All permanent Australian residents are entitled to free public hospital care when admitted to hospital as public patients. Public hospital costs are the responsibility of state and territory governments, while most community-based services are funded by the national government.

## Methods

The completed pilot included 35 case conferences involving 33 GPs between November 2011 and August 2014. The methods for the study are described elsewhere [6] but, briefly, we identified patients with end stage heart failure or non-malignant lung disease (Table 1). We conducted a single case conference involving a palliative care consultant (GM), a case management nurse specialising in heart failure or end stage respiratory disease and the patient’s GP to develop a care plan for their final months of life. There were 35 patients (26 heart failure, 9 lung disease), 21 males (60 %) with a median age of 74 years (range 61–89). The case conferences were conducted by GM, a dual general practice and palliative care specialist. Ten nurses (six heart failure, four respiratory) and 30 GPs took part (five GPs had two case conferences each).

**Table 1:** Case conference intervention

Detials of intervention	
Patients (61 to 89 years) of a regional general health service with heart failure or chronic lung disease [6] were identified as being at risk dying in the foreseeable future (usualy months), using the ‘surprise question’ [14]. The intent was not to predict prognosis, but to predict escalating need because these patients had a relapsing and remitting course. All patients were under the continuing care of a hospital based cardiologist or respiratory physician. These patients remained decompensated despite being on maximal therapy. The objective was to review the patient from a palliative perspective, plan for expected deterioration, and be ready regardless of when it would happen. A case conference between the Health service nurse/nurse practitioner, the patient’s GP and a palliative care specialist was convened to generate a clinical care plan. This plan aimed to identify patient and carer needs across clinical, personal and practical domain, and used the PEPSI COLA framework from the British Gold Standards Framework to ensure all domains were considered [15]. The nurse discussed the patient and carer needs with them and represented their views at the conference. Both nurse and and GP prepared for the meeting by reviewing the case notes. Case conferences took place either in the GP surgery or by teleconference. The Palliative care specialist acted as the conference chair and provided advice as needed. He did not see the patient unless that was an element of the plan. A written plan was generated which identified issues, what had to be done and the clinician responsible. The plan was reviewed by the patient and carer and the nurse, and endorsed or modified before being actioned. All participants, the patient and carer received a copy.	

We obtained data on hospitalisations using Australian Refined Diagnosis-Related Group (AR-DRG) codes. This is an admitted patient classification system that provides a clinically meaningful way of relating the number and type of patients treated in a hospital to the resources required by the hospital. We extracted the costs for hospitalisations from reports published by the Department of Health [8] for each corresponding financial year (2010–11 to 2014–15). We estimated the potential savings per annum from the reduced hospitalisations by comparing the period before and after the case conference (now referred to as groups) based on the time of hospital admission relative to the case conference date. We calculated the average total cost per patient for each group separately by multiplying the average number of hospital admissions (annualised) by the average cost per hospital admission. The average number of admissions was the mean of admission rates from two admission categories: ED visits leading to hospitalisation and ED visits not resulting in hospitalisation admissions [6]. The average cost per hospital admission was the product of the mean patient length of stay (PtLOS) and average cost per day. The data spanned five years, so different costs were used for each AR-DRG in each year. To account for this difference the average cost per day for each group was calculated as the mean of average cost per day estimates for each year. The average cost per day for each individual year was calculated by dividing the total average cost per AR-DRG divided by the corresponding average length of stay (ALOS, given in the AR-DRG reports). The mean cost per day estimates from 2010–2011 to 2014–2015 were used to derive the average cost per day for each group separately. Similarly the average PtLOS was derived from the mean PtLOS for each year for each group. The cost savings were determined as the difference in the average annualised cost of hospital admissions for the before and after the case conference groups, minus the average cost of conducting the case conference. The cost savings were also presented with the average total costs per patient using the admission rates excluding one service utilisation outlier from the analysis due to substantially higher utilisation of health services relative to other study participants.

We also estimated the actual cost savings per patient by multiplying the mean time from case conference to death or study closure. We then took the annualised savings per patient, calculated the daily savings by dividing this figure by 365, then multiplying that figure by the average time in the project (along with 95 % CIs).

We estimated the cost of conducting a case conference using a micro-costing approach to derive the unit cost of each resource used. The resources included the staff contributions, clinic time for the specialist, general practitioner (GP) and nursing staff, and communication costs including telephone, internet and administration time. We costed the specialist consultation using the Medicare Benefits Schedule (MBS) item number 3040 [9] for organising and co-ordinating a community case conference. We valued the specialist’s time for additional activities (including confirmation of patient suitability for CC and writing the post-CC report) using hourly wages from Queensland Health [10]. We estimated the costs for the general practitioner consultation using MBS item numbers 729 and 750 for GP management plan and team care arrangements and participating in a case conference or for contributing to a care plan in the community health team subsequent to the case conference. We costed the nursing staff time (case conference, identification of patients, preparation of pre-CC report and explaining the specialist-generated post-CC report to the patient) using hourly wages [10]. The administration costs included the time for patient recruitment (patient information, consent), notification of case conference team, case conference technical checks at the hospital site and GP practice site and report distribution post CC. The overhead resources included the postage and communications (telephone and internet) which we costed s using cost per call and cost per data unit used per CC based on costs from a national service provider [11]. We calculated the savings in two ways: i) savings from reduced hospitalisations; and ii) savings from reduced hospitalisations after deducting the costs for the case conferences. All calculations are in Australian dollars. The exchange rate averaged over the time period from January 2010 to December 2015 was AUD$1 = €0.7209 and AUD$1 = GBP£0.5912 (http://www.rba.gov.au/statistics/historical-data.html).

## Results

There were 190 hospitalisations corresponding to 63 specific AR-DRGs for the 35 patients who had case conferences. The average length of stay was 7.3 days before case conferences and 6.8 days after the case conference. The average cost of a case conference per patient was AUD$979. The specialist consultation was costed at $345 per case conference using a combination of MBS item numbers and hourly wage rates. The general practitioner consultation cost and nursing staff costs were estimated at $159 each per case conference. The administration costs were the most expensive resource at $312 and the overheads the least expensive resource at $4 per case conference. The median duration in the project for patients after case conference was 196 days (range 48 to 429). The duration was either time from case conference to death or to study close.

The average annualised number of hospitalisations was 8.9 per annum before CC and 1.3 per annum after CC [6]. The average cost per hospitalisation was AUD$10,119 before CC and AUD$9473 after CC (Table 2), a difference of AUD$646 per hospitalisation. The annualised total cost per patient was AUD$90,060 before CC and AUD$11,841 after CC. Taking into account the variable time to death among the study participants, we calculated the cost savings using the mean time in study after CC and cost saving per day inclusive of cost per case conference to be AUD$41,023 per patient after deducting the cost of case conference per patient (AUD$979, 1.2 % of the savings per patient) and AUD$25,274 per patient after excluding the service utilisation outlier.

**Table 2:** The cost of hospitalisations before and after case conference (costs in AUD$)

	Full results			Excluding service utilisation outlier		
	Before CC (Range)	After CC (Range)	Difference (95 % CIs)	Before CC (Range)	After CC (Range)	Difference (95 % CIs)
All hospitalisations	88	105		82	90	
Average patient length of stay, days	7.3 (4.7; 9.2)	6.8 (3.9; 13.2)	0.5 (−2.0; 3.0)*	7.3 (4.7; 9.2)	6.7 (3.6; 13.2)	0.6 (−1.8; 3.0)*
Average cost per day, $	$1386 (1353; 1415)	$1393 (1312; 1483)	-$7 (54.0; −68.0)*	$1357 (1266; 1432)	$1411 (1312; 1483)	$-54 (5.0;-113.0
Average cost per hospitalisation, $	$10,119	$9473	$646	$9890	$9502	$388
Average no. of hospitalisations	8.9 (3.9; 13.9)	1.3 (0.4; 2.1)	7.7^a^	6.0 (9.7; 2.3)	1.1 (0.5; 1.7)	4.9^b^
			Savings			Savings
Average total cost per patient, $	$90,060	$11,841^c^	$78,218	$59,342	$10,453	$48,890
Cost saving per day, $	$214			$134		
Cost saving per patient, $	$41,023			$25,274		

*Bolded text indicates statistically significant difference, *p* < 0.05

^a^86 % reduction

^b^82 % reduction

^c^Including AUD$979 per case conference

If there were 100 patients with end of life heart failure and lung disease each year, the case conferencing intervention would save AUD$4.1 million (and AUD$2.5 million excluding the service utilisation outlier). We attribute these savings to the reduced hospitalisation rates (79 %) and, to a lesser extent, the shorter length of stay (0.5 days) and reduction in average cost per hospital admission (AUD$646) after CC compared to before CC. Using a conservative estimate of 25 % reduction in admission rate, the cost saving per patient would be AUD$30,143 and nearly AUD$3 million per 100 patients per year.

## Discussion

The ageing of the population means that the number of deaths to be managed will rise substantially. Most will not die of cancer. The majority will not be managed by specialist palliative care services but rather by a combination of primary care and organ-based specialists. The current specialist system is aimed at acute, curative care. This pilot study examined the effect of integrating the care of life limiting heart failure and lung disease between primary and secondary care. Such patients have a highly unpredictable course. However, at some point a relapse is inevitable. The objective of the case conferences was to minimise chronic symptoms, intervene to maximise patient and carer quality of life. Finally, it empowered the patient and carer by giving them clear strategies to implement at the onset of expected deterioration. This form of care reduced the number and associated costs of hospitalisations in patients with end stage heart failure or lung disease who usually have a prognosis of survival measured in months.

There are four main limitations of our study. Firstly, the non-randomised study design precludes attribution of the savings to the case conference. Secondly, the study was conducted in one centre, which limits generalisation of the results to all hospitals. Third, the small numbers of patients and hospitalisations introduces the possibility that the sample was not representative of all people with end stage heart and lung disease. Finally, this study was not designed to not provide comprehensive costs of the ongoing care in the community. We ameliorated the changes in AR-DRG costs over time by using AR-DRG costs corresponding to the year of the event. Furthermore, we included only recurrent costs for case conferencing and excluded capital costs of equipment. We anticipate that most doctors would have access to a computer and the internet in usual practice.

Heart failure is life-threatening and predominantly affects older Australians, usually associated with poor survival. In 2007–08, there were 45,212 hospitalisations for heart failure (HF) in Australia. The average length of admission with heart failure was 8.9 days, and 8 % of all deaths in hospital were due to HF. [12] Chronic obstructive pulmonary disease was the reason for 58,900 hospital admissions in 2013–14. It was the fifth leading cause of death in Australia in 2013, accounting for 6462 (4.4 %) of all deaths [13].

One of the policy implications of this model is the potential impact of cost-shifting- the practice of transferring the caring and therefore the cost from one level of government to another. The actual cost of running a case conference in this pilot was mostly borne by staff of the hospital (state government). Case conferences were costly in terms of administration and clinical time. The economic benefits to the hospital (in reduced hospitalisations) appear to far outweigh the costs. The current funding model of hospitals, however, is activity based, so to transfer ‘clinical activity’ to the community may actually see the hospital lose funding. This may be a reason that the case conference model has not already been adopted.

If a robust trial confirms these results, the benefits of this intervention will be considerable. Despite the estimated savings, we acknowledge that there would be a major cost associated with such a large-scale health service delivery reform. Further research into community costs post-case conference is required to provide a comprehensive cost effectiveness analysis.

## Conclusions

Multidisciplinary case conferences that promote integrated care among specialists and GPs resulted in substantial cost savings while providing care. Cost shifting between national and state or territory governments may impede implementation of this successful health service intervention. An integrated model such as ours is very relevant to initiatives to reform national health care.

## Abbreviations

ALOS: Average length of stay
AR-DRG: Australian Refined Diagnosis-Related Group
AUD: Australian dollar
CC: Case conference
CI: Confidence interval
COPD: Chronic obstructive pulmonary disease
ED: Emergency department
GP: General practitioner
HF: Heart failure
MBS: Medicare Benefits Schedule
PtLOS: Patient length of stay
